# The Oneness of Disease

**Published:** 1872-09

**Authors:** 


// HALL'S
### JOURNAL OF HEALTH.

VOL. XIX. — SEPTEMBER, 1872. — NO. IX.

### THE ONENESS OF DISEASE,

And the oneness of medicine as a remedy, are well worth the attention of the unprofessional reader, and parents especially. If a man of average intellect is advised to "take something" for his ailment, and it does him good, cures him, — anybody's pills, or any old woman's tea, — he is enthusiastic in its praise. This enthusiasm increases with every experience he has of its unmistakable efficacy. It will not be long before he finds himself thinking that if it has done him good in one disease, it may do him good in another, although apparently a very different malady. Lo, and behold, it cures that too, and in a short time you find the man advising his favorite as a cure for everybody and everything. He treasures up in his own mind every case of success, and at length begins to think of setting up a patent medicine shop. Many have done it, and have made fortunes. Outsiders wonder why the doctors didn't find this out long ago, and every such person begins to feel, if he does not say, "I believe I know about as much as any of the doctors."

One half dozen medicines, skillfully employed, will cure more diseases than all the million others that are in the world. Nature will cure half of all the ailments of humanity, if you will only let her alone. The tincture of thyme (time!) will cure half the remainder. But when people get sick, they won't "take time;" they tell the doctor they can't afford it; they must take medicine, and get well right away. A day's loss to a working man, or a struggling widow, means nothing to eat on the morrow. A pill can be named — but we won't do that. If you are really sick, go to a good doctor, hand him a fee, get his advice, and follow it. Don't hook an opinion out of him, and then slip off without pay. This pill has been in use fifty years, and it will cure fifty different diseases, aches, and pains. Its composition is known to all educated men. It is so common, that many druggists keep them on hand ready made.

If a well-made machine has a thousand wheels, more or less connected in their movements, and you stop one, you stop all. The human body contains a great many glands, manufactories, and wheels. Stop the great one, the heart, and you stop all. "Slow" one, and you "slow" all. Put upon one organ a thousand horse-power extra, and make it run a mile a minute, such as a tremendous dose of brandy, and the other organs will run like lightning.

The liver is one of the great controlling organs of the body. Regulate its working healthfully, and the other parts of the machine dependent on it will run healthily too. And in some way or other, almost every part of the human body has a more or less direct connection with the liver, sympathizing with its action. Hence, any medicine acting on the liver so as to restore it to a healthy working, necessarily restores all the other parts which depend on it.

The pills in question do affect the liver, and are called "Purgative Pills," or liver pills, or any other name that suits the whim of the enterprising inventor of the same, who avers they are infallible, and may be given to advantage in all forms of disease which are associated with constipated bowels, with fevers, with headache, with cold feet, foul tongue, variable appetite, nausea, or pain.

Two or three should be taken at a time, and if they do not operate within twelve hours, then take a tablespoon of castor oil, or as much salts in a glass of warm water every hour until there is a discharge from the bowels ; or an injection may be taken instead of the oil or salts, although it will not be as efficient in its action.

The pills should be made to act as above, because they contain a small amount of calomel, which may now and then cause salivation, if it is allowed to remain too long in the system. But these pills contain ingredients which usually have the effect to act on the stomach and bowels, irrespective of the calomel, thus making it unnecessary to take anything after them to work it off.

It is not advised to take them oftener than once in five or six days, as, if taken at too short intervals, they might salivate. They are intended to act on the liver, to thin the blood, to lessen its quantity, to improve its quality, to make it good and life-giving.

FEVERS

of all descriptions will be benefited and cured by the judicious administration of these pills from time to time, until the patient begins to feel as if bread and butter and roasted meat would taste very good. No other medicine is really needed during the taking of these pills ; the system should be allowed to rest. If every trifling symptom is prescribed for, it will be found in a short time that some kind of medicine is to be taken almost every hour of the day and night, until outraged nature begins to reject everything, and scarcely tolerates a glass of the purest water.

The system will sooner rectify itself if left alone to the recuperative powers of nature, these pills being given merely to aid her a little in clearing the liver of the obstructions in it, so that the blood may pass on readily to other parts of the system. Each person should note how many are required to cause one operation within twelve hours, for some are more sensitive to the action of medicine than others, and a motion may be more urgently needed at one time than at another. In such cases it would be better to take an additional pill, in order to be more certain of its effect, and that this effect should be more full and free.

### LOOSE BOWELS.

Two or three of these pills will cure any ordinary case of loose bowels, if the patient will go to bed at once, wrap up warm, and eat nothing for a day or two but boiled rice, with a small amount of boiled milk, using no other liquid whatever. But in taking these pills for diarrhœa, which is the physician's name for loose bowels, if they do not stop the passages within two hours, it is proof that a large enough dose has not been taken. Hence, at the end of two hours, if the bowels are still running off, many double the dose; if three were taken, take five, and pay more attention to giving the body rest, and keeping it warm.

These pills will keep good in a dry place for twenty years. They contain no dangerous ingredients; and if all other medicines are abstained from, and cleanliness, and pure air, and warmth, and quiet are secured, with a moderate diet, there is scarcely a disease of any ordinary character which will not be benefited by them, for the simple reason that they will always do more or less towards purifying the blood, lessening its quantity, and giving it a more healthful circulation. With these, a person may travel the world over without the need of any other remedy to supplement his medicine chest; because, in the large majority of ordinary attacks of sickness, quiet, warmth, rest, pure air, and abstinence from all food for twelve or twenty-four hours, will inaugurate an immediate improvement, which would result in cure, if the means stated were persevered in. At the end of fifteen or twenty hours after taking the dose, the patient should eat something, if there is a decided feeling of hunger; if not, wait for it, even if it is for three or four days, taking care not to eat at shorter intervals than five hours, eating moderately of only two kinds of food, bread being one.

But, although this is incomparably the best course to pursue, there are circumstances which make it necessary to have more immediate relief. Thus, at the first attack of sickness, if the bowels have not acted freely within twenty-four hours, take an emetic; if this is not convenient, then take three of the pills, and await their action.

If within three or four hours after taking the pills there is an action of the bowels, it is not from the pills; hence, if at the end of twelve hours there is not an action of the bowels, bring about such action, although they were moved within four hours after the pills were taken.

It is important to know if the pills are working favorably, which is ascertained by the color and consistence of the discharges from the bowels. If the discharges are large in amount, and somewhat thicker or more consistent than water, if they are very offensive, if they are a light, bright yellow, the pills have done good, but more if they are green, and more still if they are dark or black. Sometimes they are described as being

"BLACK AS TAR,"

these being indications of the different degrees of torpidity, or congestion of the liver. Hence, whatever medicines persons take, the discharges from the bowels afterwards should be scrutinized, to be able to decide on the beneficial effects of the medicine, if no physician is in attendance, and for his satisfaction, if you have one.

Sometimes nature herself throws off very yellow, green, or black discharges, thus showing that she has vigor enough to relieve herself of the diseased conditions of the system; and although these passages may be free and frequent, if they are of greater consistence than water, it is a

HEALTHFUL DIARRHŒA, and nothing ought to be taken to arrest it; but this is often ignorantly done, to serious, and sometimes fatal injury, under the impression that it is a diseased condition, hurtful to the system, when in reality it is an effort of nature to cure herself. In the case, however, of green, flaky passages from infants, means should be taken to correct them. The pills usually act in about ten or twelve hours, and then again once or twice during the day. In proportion as the discharges are thin, light-colored, and weakening, the pills have failed of their proper effect. Then more attention should be paid to warmth, rest, abstinence from food, unless hungry; then take boiled rice with boiled milk in small quantities; have a stout woolen flannel bound very tightly about the abdomen, some fourteen inches broad and long enough to have a double layer in front. If the passages still continue, keep quiet, and wait. Nothing more will be needed.

The value of these purgatives will be more intelligently appreciated by a simple enumeration of the diseased conditions to which they are applicable, the most of these symptoms being more or less directly associated with a torpid condition of the liver, although it is rare that more than two or three are present in any one case at a time.

| | |
|---|---|
| Appetite Variable, | Flashes of Heat, |
| Bad Taste, | Flatulence, |
| Belching, | Fullness of Stomach, |
| Bitter Taste, | General Distress, |
| Brownish Spots, | Gloominess, |
| Chilliness, | Headache, |
| Choking Sensation, | Heaviness at Stomach, |
| Cold Feet, | Internal Heat, |
| Colic, | Low Spirits, |
| Constipation, | Nervousness, |
| Despondency, | Numbness, |
| Diarrhœa, | Pain in Back, |
| Discouragement, | Pain in Breast, |
| Dizziness, | Pain in Bowels, |
| Drowsiness, | Pain in Shoulders, |
| Dry Cough, | Pain in Sides, |
| Dry Throat, | Palpitation, |
| Dullness, | Piles, |
| Dyspepsia, | Raising Blood, |

| | |
|---|---|
| Rush of Blood, | Sore Throat, |
| Sallow Skin, | Unnatural Urine, |
| Sore Bowels, | Yellow Skin. |

It would be interesting and instructive to point out how these various symptoms may and do arise from a disordered condition of the liver, but want of space forbids.

In addition to all these ailments, which are directly reached by the purgative pills, there is scarcely a pain or an ache in the whole human body which will not be more or less modified by one full, free, thorough operation upon the system. Moreover, they will cure almost any curable case of

FEVER AND AGUE; will break up the chills in a single day, sometimes, although of many months' duration, by simply taking enough of them two hours before the expected chill. If four of them are taken one day, and the chill is not broken, is not prevented from occurring, take a double dose next time. The philosophy of this action is, that the pills at once begin to excite an activity in the circulation, which compels a more lively flow of the blood, throws it out from the centres of the body to its circumference, and thus prevents that stagnation, that congestion of the blood, which is the immediate cause of the chill. Then if the chill is prevented, there is no reaction to cause a fever, and its consequences of debilitating perspiration. To make the matter sure, take another dose two hours before the next expected chill, and the work is done, but with much greater certainty if, when the pills begin to act on the bowels, about three grains of quinine are taken in water every two hours until the time for the chill of the next day has passed, omitting the quinine after ten o'clock at night until daylight next morning, so as to allow the patient to sleep, the diet meanwhile being on the general principles of three meals a day, at not less than five hours' interval, of bread and butter and meat at breakfast and dinner, and at supper bread and butter only, with a cup of hot tea.

Here, then, is a pill of such constituents as will make it act on the stomach, liver, and bowels; and, doing so, will modify all the symptoms which arise from their deranged condition.

It sometimes happens that a constipated condition of the bowels will give rise to forty or fifty different symptoms in as many different persons at least, not more than two or three, if any of them, found in the same persons; and an ignorant mind, noticing the results in this connection, could be very easily led to attribute to this pill a miraculous power; but it is in reality a simple thing; it is one medicine producing a wide-reaching effect. But it is easy to see that any other means which would clear the system of its accumulations would give the same good results.

A due consideration of the subjects mentioned will serve to show reflecting persons that there is a oneness in disease, and in its remedy, which vastly simplifies the practice of medicine, and which removes from it much of the mystery which hangs around it in the minds of a large class of the community. And a proper reflection on the whole matter leads to the legitimate conclusion, that the practice of medicine, in the hands of educated men of experience and skill, is more of a science than most persons think it is.

